# Time Trends in Use of Radical Prostatectomy by Tumor Risk and Life Expectancy in a National Veterans Affairs Cohort

**DOI:** 10.1001/jamanetworkopen.2021.12214

**Published:** 2021-06-03

**Authors:** Kristina Vaculik, Michael Luu, Lauren E. Howard, William Aronson, Martha Terris, Christopher Kane, Christopher Amling, Matthew Cooperberg, Stephen J. Freedland, Timothy J. Daskivich

**Affiliations:** 1 Los Angeles County Department of Public Health, Los Angeles, California; 2 Cedars-Sinai Center for Outcomes Research and Education, Los Angeles, California; 3 Divison of Urology, Cedars-Sinai Medical Center, Los Angeles, California; 4 Section of Urology, Durham Veterans Affairs Medical Center, Durham, North Carolina; 5 Division of Urology, West Los Angeles Veterans Affairs Medical Center, Los Angeles, California; 6 Department of Urology, University of California at Los Angeles School of Medicine, Los Angeles; 7 Divison of Urology, Charlie Norwood Veterans Affairs Medical Center, Augusta, Georgia; 8 Section of Urology, Medical College of Georgia, Augusta; 9 Urology Department, University of California at San Diego Health System, San Diego; 10 Department of Urology, Oregon Health & Science University, Portland; 11 Department of Urology, University of California at San Francisco Helen Diller Family Comprehensive Cancer Center, San Francisco

## Abstract

**Importance:**

Guidelines endorse using tumor risk and life expectancy (LE) to select appropriate candidates for radical prostatectomy (RP), recommending against treatment of most low-risk tumors and men with limited LE.

**Objective:**

To investigate time trends in the use of RP by tumor risk and Prostate Cancer Comorbidity Index (PCCI) score in a contemporary, nationally representative Veterans Affairs (VA) cohort.

**Design, Setting, and Participants:**

This cohort study of 5736 men treated with RP at 8 VA hospitals from January 1, 2000, to December 31, 2017, used a nationally representative, multicenter sample from the VA SEARCH (Shared Equal Access Regional Cancer Hospital) database. Statistical analysis was performed from June 30, 2018, to August 20, 2020.

**Main Outcomes and Measures:**

Stratified linear and log-linear Poisson regressions were used to estimate time trends in the proportion of men treated with RP across American Urological Association tumor risk and PCCI (a validated predictor of LE based on age and comorbidities) subgroups.

**Results:**

Among 5736 men (mean [SD] age at surgery, 62 [6] years) treated with RP from 2000 to 2017, the proportion of low-risk tumors treated with RP decreased from 51% to 7% (difference, −44%; 95% CI, −50% to −38%). The proportion of intermediate-risk tumors treated with RP increased from 30% to 59% (difference, 29%; 95% CI, 23%-35%), with unfavorable intermediate-risk tumors increasing from 30% to 41% (difference, 11%; 95% CI, 4%-18%) and favorable intermediate-risk tumors decreasing from 61% to 41% (difference, −20%; 95% CI, −24% to −15%). The proportion of high-risk tumors treated with RP increased from 18% to 33% (difference, 15%; 95% CI, 9%-21%). Among men treated with RP, the proportion with the highest PCCI scores of 10 or more (ie, LE <10 years) increased from 4% to 13% (difference, 9%; 95% CI, 4%-14%). Within each tumor risk subgroup, no significant difference in the rate of tumors treated with RP over time was found across PCCI subgroups.

**Conclusions and Relevance:**

In this study, the use of RP shifted from low-risk and favorable intermediate-risk to higher-risk prostate cancer. However, its use among men with limited LE appears unchanged across tumor risk subgroups and increased overall.

## Key Points

**Question:**

How has the use of radical prostatectomy (RP) changed over time with respect to tumor risk and life expectancy (LE)?

**Findings:**

In this cohort study of 5736 men treated with RP at 8 Veterans Affairs hospitals from 2000 to 2017, the proportion of low-risk tumors decreased 44%, the proportion of intermediate-risk tumors increased 29% (with favorable intermediate-risk tumors decreasing 20% and unfavorable intermediate-risk tumors increasing 11%), and the proportion of high-risk tumors increased 15%. During this period, the proportion of men treated with RP with LE less than 10 years increased 9%.

**Meaning:**

These findings suggest that although use of RP has shifted away from low-risk and favorable intermediate-risk to higher-risk prostate cancer, its use among men with limited LE appears unchanged across tumor risk subgroups and increased overall.

## Teaser

This cohort study investigates time trends in use of radical prostatectomy by tumor risk and Prostate Cancer Comorbidity Index score in a contemporary, nationally representative Veteran Affairs cohort.

## Introduction

With the endorsement of active surveillance for low-risk and favorable intermediate-risk prostate cancer (PCa) by guideline-producing bodies during the last 10 years,^1,2,3^ active surveillance has gradually replaced radical prostatectomy (RP)^4^ as the most common treatment approach for low-risk PCa. Concurrent increases in the use of active surveillance, or conservative management, and decreases in the use of RP for low-risk disease have been observed across numerous practice settings.^4,5,6^ These trends indicate that physicians and patients are appropriately paying greater attention to tumor risk before proceeding with potentially morbid local therapy, such as surgery or radiotherapy.

Guidelines also universally highlight the importance of life expectancy (LE) when considering aggressive vs conservative management for nonmetastatic PCa. In fact, the National Comprehensive Cancer Network guidelines^1^ use LE as the primary branch point in triaging between aggressive and conservative management for all tumor risk subtypes. Considering that survival benefits associated with aggressive local therapy do not manifest until about 10 years after surgery^7^ and treatment often worsens quality of life in older men and men with more severe illness,^8,9^ National Comprehensive Cancer Network guidelines recommend observation or nonsurgical management for men with less than 10 years’ LE and low-risk or intermediate-risk PCa.

Although epidemiologic studies have documented increasing use of active surveillance among subgroups of tumor risk and age,^4,5,6,10,11,12,13^ few studies have explicitly looked at treatment trends by LE at diagnosis, to our knowledge. Although age can serve as a rough proxy for LE, both age and comorbidity have been shown to be independently associated with LE^14^; therefore, both should be factored into clinical treatment decisions. Furthermore, several studies have shown that overtreatment of men with limited LE occurs most often in men with more severe illness, not older men.^15,16^ Therefore, in this study, we sought to define treatment trends in the use of RP by tumor risk and LE in the active surveillance era using the Prostate Cancer Comorbidity Index (PCCI), a weighed scale based on age and comorbidity that was created and validated in men with PCa to predict long-term noncancer mortality (ie, LE).^17^ Among those treated with RP, men with PCCI scores of 0 to 2, 3 to 6, 7 to 9, and 10 or higher have 10-year overall mortality rates of 7% to 15%, 19% to 28%, 41%, and 51%, respectively.^18^ In this study, we analyzed the individual association of LE and tumor risk with time trends in the use of RP among subgroups of men with different PCCI scores (0-2, 3-6, 7-9, and ≥10) and tumor risk (very low, low, intermediate, favorable intermediate, low-volume favorable intermediate [≤2 positive biopsy cores], unfavorable intermediate, and high). We hypothesized that LE would have a modest association with the use of RP over time compared with tumor risk.

## Methods

### Data Source and Analytic Sample

We sampled 5736 men treated with RP for nonmetastatic PCa between January 1, 2000, and December 31, 2017, from the Shared Equal Access Regional Cancer Hospital (SEARCH) database, which includes data from 8 Veterans Affairs (VA) hospitals across the United States (West Los Angeles, California; Palo Alto, California; San Diego, California; San Francisco, California; Augusta, Georgia; Durham, North Carolina; Asheville, North Carolina; and Portland, Oregon). Of the 5736 men in the analytic sample, 614 (11%) were missing data on 1 or more of the study variables. Missing data rates were assessed for race/ethnicity (0.5% [27 of 5736]), D’Amico risk (0.5% [29 of 5736]), clinical stage (0.1% [7 of 5736]), Gleason score (0.4% [25 of 5736]), prostate-specific antigen (PSA) level (0.04% [2 of 5736]), number of positive cores (9% [510 of 5736]), percentage of positive cores (11% [621 of 5736]), and PSA density (17% [964 of 5736]). To reduce the potential for bias, missing data were imputed with the multiple imputation by chained equation algorithm.^19^ Relevant VA and Cedars-Sinai internal review boards approved this study. This retrospective cohort study was conducted under a waiver of informed consent and Health Insurance Portability and Accountability Act authorization. Consent was waived because the research was minimal risk and could not practicably be carried out without the waiver, as many of the patients were already deceased. This study complied with the Strengthening the Reporting of Observational Studies in Epidemiology (STROBE) reporting guideline for observational studies.^20^

### Variable Definitions

#### Tumor Risk

Tumors were stratified into low-, intermediate-, and high-risk categories using American Urological Association tumor risk criteria.^21,22^ Intermediate-risk tumors were further stratified into 3 mutually nonexclusive subgroups: favorable intermediate risk, low-volume favorable intermediate risk, and unfavorable intermediate risk. Favorable intermediate risk was defined as tumors with 1 intermediate-risk factor (PSA level, 10-20 ng/mL [to convert to micrograms per liter, multiply by 1.0]; grade group 2; or clinical stage of T2b) and 50% or fewer positive biopsy cores. Unfavorable intermediate risk was defined as 2 or more intermediate-risk factors, 50% or more positive biopsy cores, or grade group 3 disease.^23^ Low-volume favorable intermediate risk was defined as tumors with 1 intermediate risk factor and 2 or fewer positive biopsy cores.

#### Life Expectancy

The PCCI is an externally validated, long-term, other-cause mortality prediction tool for early-stage PCa modeled after the Charlson Comorbidity Index.^24^ It uses age and comorbidity at diagnosis to provide a weighted numerical score predicting long-term, other-cause mortality in men with PCa. The PCCI is calculated by adding 1 point for each 6 years older than 60 years and additional points for the number and type of comorbidities, as previously described.^17^ Prostate Cancer Comorbidity Index scores were grouped a priori into 4 categories (0-2, 3-6, 7-9, and ≥10) based on similar long-term mortality rates in a prior study of men with PCa in the VA Health System.^17^

### Statistical Analysis

Statistical analysis was performed from June 30, 2018, to August 20, 2020. Patient demographic and clinical characteristics were compared across tumor risk strata (low, intermediate, and high risk) using the Kruskal-Wallis test and the Pearson χ^2^ test for continuous and categorical variables as appropriate. The primary outcome of our study was the rate of patients receiving RP over time. Patient-level data were summarized on an annual level to estimate the proportion of patients receiving RP by PCCI, tumor risk, and by PCCI × tumor risk stratification. Univariate ordinary least-squares regression was used to estimate the absolute change in the proportion of patients receiving RP by each tumor risk and PCCI stratification. Furthermore, a log-linear Poisson regression model with an offset was used to estimate the annual percentage change in the rate of RP across our study period by each PCCI × tumor risk stratification. A test of interaction between year of surgery and PCCI subgroups by each tumor risk stratification was performed to test for differences in the annual percentage change of RP over time between PCCI subgroups. A total of 21 comparisons were performed, with 3 comparisons within each tumor risk stratification. A Bonferroni-adjusted significance level of (.05/21) *P* < .002 was used to assess for significance of interactions.

We also conducted sensitivity analyses checking for interactions between race/ethnicity and year of surgery with annual percentage change of RP over time by each tumor risk stratification. We also conducted a parallel analysis with region (Northwest, Southern California, and South) instead of race/ethnicity. All statistical analyses were performed in R, version 3.5.1 (R Group for Statistical Computing)^25^ using 2-sided tests with a significance level of *P* < .05.

## Results

Among our analytic sample of 5736 men, the mean (SD) age was 62 (6) years, and most patients were White (3761 [66%]) and Black (1780 [31%]) (Table 1). Demographic, clinical, and pathologic sample characteristics stratified by tumor risk are provided in Table 1. Close to half the study sample had intermediate-risk tumors (2602 [45%]), and the remainder was split approximately evenly between low-risk (1617 [28%]) and high-risk (1517 [26%]) tumor groups. Among the 2602 patients with intermediate-risk tumors, 1287 (50%) were classified as favorable intermediate risk, 492 (19%) as low-volume favorable intermediate risk, and 928 (36%) as unfavorable intermediate risk. Men with higher tumor risk tended to be older, with greater preoperative PSA values, biopsy grade group scores, PCCI scores, and clinical stage (Table 1).

**Table 1.** Sample Characteristics

Characteristic	Patients, No. (%)				*P* value^a^
	Overall (N = 5736)	Low risk (n = 1617)	Intermediate risk (n = 2602)	High risk (n = 1517)	
Age at surgery, y					
Mean (SD)	62 (6)	61 (6)	62 (6)	63 (6)	<.001
Median (IQR)	62 (58-66)	61 (57-65)	63 (58-66)	63 (59-67)	
Race/ethnicity					
Black	1780 (31)	481 (30)	864 (33)	435 (29)	.02
White	3761 (66)	1086 (67)	1643 (63)	1032 (68)	
Asian or Pacific Islander	147 (3)	34 (2)	73 (3)	40 (3)	
American Indian or Alaska Native	48 (1)	16 (1)	22 (1)	10 (1)	
Comorbidity score, PCCI					
0-2	2723 (47)	850 (53)	1230 (47)	643 (42)	<.001
3-6	1864 (32)	537 (33)	844 (32)	483 (32)	
7-9	663 (12)	157 (10)	296 (11)	210 (14)	
≥10	486 (9)	73 (5)	232 (9)	181 (12)	
Year of surgery					
2000-2002	726 (13)	335 (21)	235 (9.0)	156 (10)	<.001
2003-2005	914 (16)	393 (24)	354 (14)	167 (11)	
2006-2008	947 (17)	348 (22)	383 (15)	216 (14)	
2009-2011	1134 (20)	297 (18)	525 (20)	312 (21)	
2012-2014	1162 (20)	149 (9)	607 (23)	406 (27)	
2015-2017	853 (15)	95 (6)	498 (19)	260 (17)	
Preoperative PSA level, ng/mL					
Mean (SD)	8.5 (8.3)	5.4 (2.1)	7.9 (4.0)	12.8 (14.2)	<.001
Median (IQR)	6.4 (4.8-9.6)	5.2 (4.2-6.8)	6.8 (5.0-10.3)	8.0 (5.4-14.8)	
Medical center					
West Los Angeles, California	751 (13)	164 (10)	329 (13)	258 (17)	<.001
Palo Alto, California	429 (8)	165 (10)	182 (7)	82 (5)	
San Diego, California	776 (14)	213 (13)	335 (13)	228 (15)	
San Francisco, California	616 (11)	133 (8)	312 (12)	171 (11)	
Augusta, Georgia	949 (17)	264 (16)	473 (18)	212 (14)	
Durham, North Carolina	841 (15)	271 (17)	411 (16)	159 (10)	
Asheville, North Carolina	540 (9)	221 (14)	206 (8)	113 (7)	
Portland, Oregon	834 (15)	186 (12)	354 (14)	294 (19)	
Biopsy Gleason sum					
≤6	2094 (37)	1617 (100)	324 (12)	153 (10)	<.001
7	2626 (46)	0	2278 (88)	348 (23)	
8-10	1016 (18)	0	0	1016 (67)	
Clinical stage					
T1	3421 (60)	1130 (70)	1666 (64)	625 (41)	<.001
T2	2293 (40)	487 (30)	936 (36)	870 (57)	
≥T3	22 (0.4)	0	0	22 (2)	

Abbreviations: IQR, interquartile range; PCCI, Prostate Cancer Comorbidity Index; PSA, prostate-specific antigen.

SI conversion factor: To convert PSA to micrograms per liter, multiply by 1.0.

^a^ Statistical tests performed: the Kruskal-Wallis test for continuous variables and the χ^2^ test of independence for categorical variables.

**Figure 1.**
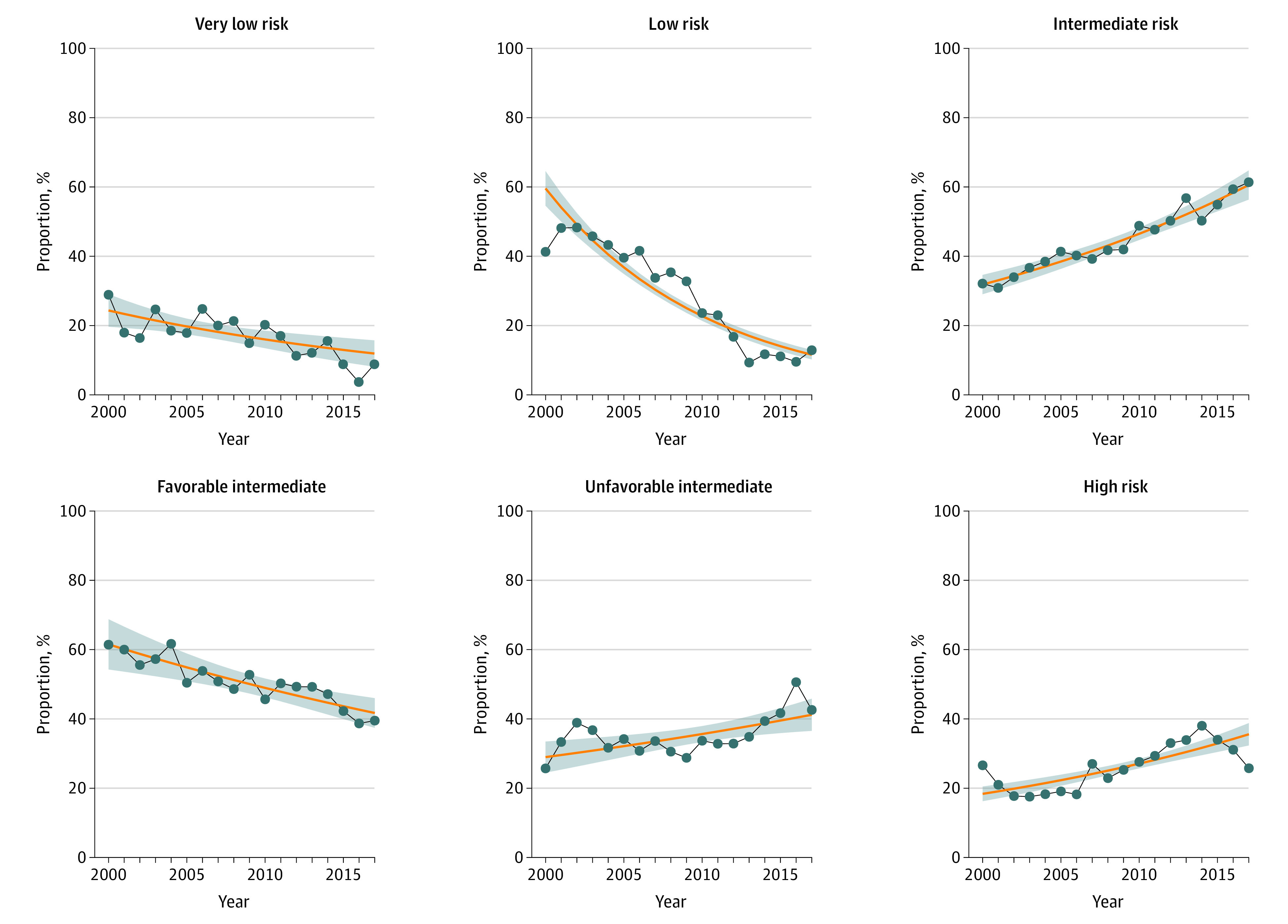
Temporal Trends in Proportion of Men Treated With Radical Prostatectomy From 2000 to 2017 by American Urological Association Tumor Risk The blue points and blue line show the trend of the proportion of tumor risk among patients undergoing radical prostatectomy. The orange trend line shows the estimated proportion of tumor risk among patients undergoing radical prostatectomy using the log-linear Poisson regression model. The shaded area represents the 95% CI of the estimates from the log-linear Poisson regression model.

When stratified by tumor risk, there was a decrease in low-risk tumors and increases in intermediate- and high-risk tumors treated with RP over the study period (Figure 1 and Table 2). Specifically, from 2000 to 2017, low-risk tumors decreased from 51% to 7% (difference, –44%; 95% CI, −50% to −38%; *P* < .001). Intermediate-risk tumors increased from 30% to 59% (difference, 29%; 95% CI, 23%-35%; *P* < .001). Within the intermediate-risk tumor category, unfavorable intermediate-risk tumors increased during the study period, whereas both favorable intermediate-tumor risk groups decreased. Unfavorable intermediate-risk tumors increased from 30% to 41% (difference, 11%; 95% CI, 4%-18%; *P* = .002). Favorable intermediate-risk tumors decreased from 61% to 41% (difference, –20%; 95% CI, −24% to −15%; *P* < .001), and low-volume favorable intermediate-risk tumors decreased from 35% to 7% (difference, –28%; 95% CI, −33% to −24%; *P* < .001). Finally, the proportion of patients with high-risk tumors treated with RP during the study period increased from 18% to 33% (difference, 15%; 95% CI, 9%-21%; *P* < .001).

**Table 2.** Absolute Percentage Change in Proportion of Men Treated With Radical Prostatectomy From 2000 to 2017 Within AUA Tumor Risk and PCCI Subgroups

Subgroup	Absolute change from 2000 to 2017, % (95% CI)	*P* value
**AUA tumor risk**		
Low	−44.1 (−49.9 to −38.2)	<.001
Very low	−15.8 (−21.9 to −9.8)	<.001
Intermediate	28.8 (22.9 to 34.7)	<.001
Low-volume favorable	−28.2 (−32.6 to −23.9)	<.001
Favorable	−19.7 (−24.2 to −15.3)	<.001
Unfavorable	11.2 (4.0 to 18.4)	.002
High	15.3 (9.4 to 21.2)	<.001
**PCCI score subgroups**		
0-2	−21.9 (−27.0 to −16.9)	<.001
3-6	8.5 (3.5 to 13.6)	<.001
7-9	4.5 (−0.5 to 9.5)	.08
≥10	8.9 (3.9 to 14.0)	<.001

Abbreviations: AUA, American Urological Association; PCCI, Prostate Cancer Comorbidity Index.

**Figure 2.**
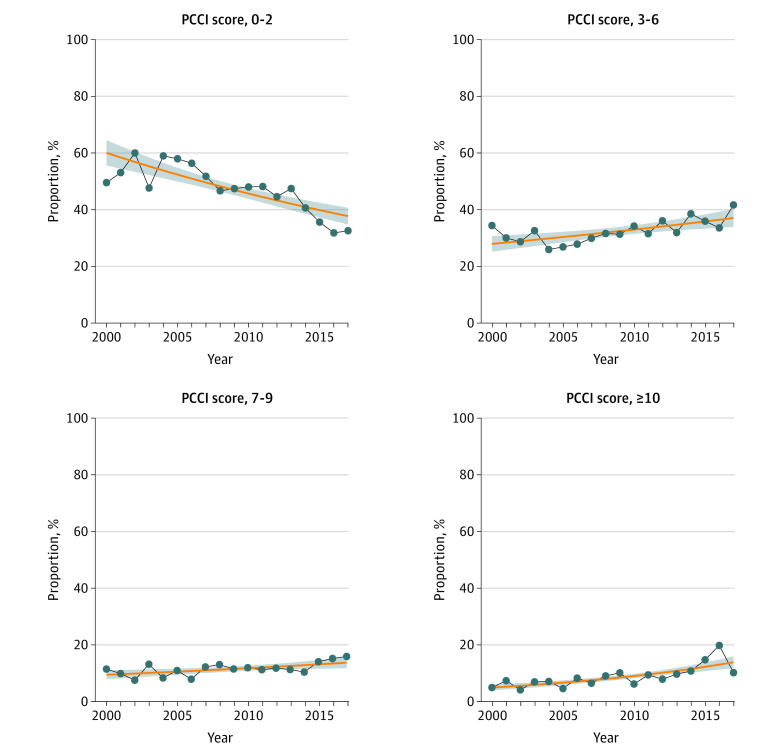
Temporal Trends in Proportion of Men Treated With Radical Prostatectomy From 2000 to 2017 by Prostate Cancer Comorbidity Index (PCCI) Score The blue points and blue line show the trend in the proportion of patients undergoing radical prostatectomy with the given PCCI score range. The orange trend line shows the estimated proportion of patients undergoing radical prostatectomy with the given PCCI score range using the log-linear Poisson regression model. The shaded area represents the 95% CI of the estimates from the log-linear Poisson regression model.

When stratified by PCCI scores, there was no decrease in the proportion treated with RP for men with the highest PCCI scores (Figure 2 and Table 2). Among men treated with RP, the proportion with PCCI scores of 7 to 9 increased from 9% to 14% (difference, 4%; 95% CI, −0.5% to 10%; *P* = .08). The proportion with PCCI scores of 10 or more increased from 4% to 13% (difference, 9%; 95% CI, 4%-14%; *P* < .001).

When stratified by both tumor risk and PCCI scores, there were no significant differences in the use of RP across PCCI scores within strata of tumor risk (eFigure in the Supplement). In other words, within each tumor risk category, there were no trends away from treating those with the highest PCCI scores with RP. Ranges of annual percentage changes in the use of RP across PCCI subgroups within the same tumor risk strata were narrow (Table 3). Tests for interaction between year of surgery and PCCI within each tumor risk subgroup were not statistically significant (Table 3). Tests for interaction between year of surgery and race or region within each tumor risk subgroup were also not statistically significant.

**Table 3.** Annual Percentage Change in Proportion of Men Treated With Radical Prostatectomy From 2000 to 2017 by PCCI Subgroup Within AUA Tumor Risk Categories

AUA tumor risk and PCCI subgroup	Annual change		Year × PCCI interaction *P* value
	Estimate, % (95% CI)	*P* value	
Very low			
0-2	−4.1 (−8.0 to 0.0)	.049	[Reference]
3-6	−2.2 (−6.3 to 2.1)	.31	.53
7-9	−0.6 (−8.8 to 8.4)	.90	.47
≥10	−1.8 (−14.6 to 12.8)	.80	.76
Low			
0-2	−8.4 (−9.7 to −7.0)	<.001	[Reference]
3-6	−8.9 (−10.4 to −7.3)	<.001	.63
7-9	−10.6 (−13.4 to −7.6)	<.001	.19
≥10	−10.9 (−14.9 to −6.7)	<.001	.27
Intermediate			
0-2	3.9 (2.6 to 5.1)	<.001	[Reference]
3-6	3.9 (2.5 to 5.4)	<.001	.93
7-9	4.5 (2.0 to 7.0)	<.001	.66
≥10	3.3 (0.5 to 6.2)	.02	.73
Low-volume favorable			
0-2	−6.4 (−8.8 to −4.0)	<.001	[Reference]
3-6	−9.5 (−12.4 to −6.6)	<.001	.11
7-9	−5.4 (−10.4 to −0.1)	.045	.71
≥10	−13.7 (−19.1 to −8.0)	<.001	.02
Favorable			
0-2	−1.9 (−3.5 to −0.3)	.02	[Reference]
3-6	−2.6 (−4.5 to −0.6)	.01	.59
7-9	−0.9 (−4.4 to 2.6)	.60	.63
≥10	−4.3 (−8.1 to −0.3)	.03	.27
Unfavorable			
0-2	1.7 (−0.3 to 3.8)	.09	[Reference]
3-6	2.7 (0.2 to 5.3)	.04	.57
7-9	−0.6 (−4.5 to 3.5)	.76	.31
≥10	3.6 (−1.2 to 8.6)	.15	.50
High			
0-2	4.1 (2.4 to 5.9)	<.001	[Reference]
3-6	4.2 (2.3 to 6.2)	<.001	.95
7-9	2.5 (−0.3 to 5.4)	.08	.34
≥10	1.4 (−1.6 to 4.6)	.36	.14

Abbreviations: AUA, American Urological Association; PCCI, Prostate Cancer Comorbidity Index.

## Discussion

The present study shows a marked decrease in the use of RP for low-risk and favorable intermediate-risk PCa, with a concomitant increase in its use for unfavorable intermediate-risk and high-risk PCa in the active surveillance era. From 2000 to 2017, there were decreases in the proportion of low-risk tumors (from 51% to 7%), favorable intermediate-risk tumors (from 61% to 41%), and low-volume favorable intermediate-risk tumors (from 35% to 7%) treated with RP. This change was compensated for by an increase in the use of RP for unfavorable intermediate-risk tumors (from 30% to 41%) and high-risk tumors (from 18% to 33%). However, despite these drastic differences in management by tumor risk, there was minimal to no change in the proportion of men treated with RP by LE within each tumor risk category. These findings suggest that, while tumor risk has been playing a greater role in determining appropriate use of RP—particularly for men with low-risk and favorable intermediate-risk disease who are ideal candidates for conservative management—the incorporation of LE in the treatment decision-making process remains an issue and is associated with the persistent overtreatment of men with limited LE.

The observed trends in the use of RP by tumor risk are consistent with observations by other groups in and outside of the VA setting. Since the early 2000s, a concurrent shift toward increasing conservative management^4,26^ and decreasing aggressive treatment of low-risk PCa has been observed in both the US^5,6^ and internationally.^11^ A retrospective study investigating treatment trends among a national sample of men from the Cancer of the Prostate Strategic Urologic Research Endeavor (CaPSURE) study found a marked increase in the active surveillance of patients with a low Cancer of the Prostate Risk Assessment (CAPRA) score alongside decreases in the use of aggressive treatments, including RP, from 2010 to 2013.^5^ Similarly, a recent retrospective study investigating PCa treatment trends from 2000 to 2015 among a national sample of men from the Surveillance, Epidemiology, and End Results (SEER) Prostate Active Surveillance/Watchful Waiting Database found an increase in the use of active surveillance and watchful waiting alongside a decrease in the use of RP to treat low-risk disease.^6^ Although both of these studies illustrate a shift from aggressive to conservative treatments in groups with low-risk tumors, they do not investigate changes in the tumor risk composition of patients receiving RP or by more granular assessments of tumor risk, specifically favorable intermediate-risk and low-volume favorable intermediate-risk disease. However, the present and aforementioned studies are consistent; all 3 studies observed a decrease in the use of RP among low-risk tumors and either a stable or increased use among intermediate-risk and high-risk tumors.

However, our study also shows that there has been very little change in treatment patterns of patients undergoing RP by LE during the same observation period. Despite overall reductions in the proportion of low-risk, favorable intermediate-risk, and low-volume favorable intermediate-risk tumors being treated aggressively with RP, the absence of a statistically significant difference in these trends among patients in different PCCI subgroups—and the absence of a considerable decrease in those with the highest PCCI scores—suggests that LE may be playing a lesser role in the treatment decision-making process, resulting in consistent overtreatment of men with limited LE. The shared decision to pursue aggressive treatment despite limited LE may be owing to factors associated with physicians (eg, failure to consider LE and inadequate communication of LE in treatment consultations) and factors associated with patients (eg, skepticism of LE prediction accuracy and hope for a longer LE), and the available data cannot parse the reason for overtreatment. However, given the low risk of cancer mortality and high risk of other-cause mortality among men with limited LE and lower-risk disease, it is concerning that there are not more drastic changes in the proportion of patients treated with RP over time. For example, it is surprising that there was not more of a decrease in the use of RP for those with favorable intermediate-risk disease and limited LE; despite nonsignificant trends toward greater decreases in RP among those with limited LE for low-risk and low-volume favorable intermediate-risk disease, all PCCI subgroups had similar relative decreases over time for favorable intermediate-risk disease (range, −0.9% to −4.3%). Furthermore, unlike the low-risk and low-volume favorable intermediate-risk groups that reached a nadir of approximately 10% of those treated in 2017, the favorable intermediate-risk group still was at approximately 35% to 40% of men treated with RP in 2017 across all PCCI subgroups, so the lack of differences between PCCI subgroups is not mediated by a floor effect (eFigure in the Supplement).

It is also surprising that there is not a statistically significant temporal increase in the proportion of men with limited LE being treated for high-risk disease in line with the trend in the overall population; for example, for men with a PCCI score of 10 or more, the annual percentage change in the treatment of high-risk disease was only 1% (95% CI, −2% to 5%; *P* = .36) compared with 4% (95% CI, 5%-9%; *P* < .001) for all patients. Such an increase would have been consistent with guidelines recommending aggressive treatment at a lower LE threshold for high-risk disease compared with lower risk disease (ie, LE of >5 years for the high-risk subgroup vs >10 years for other risk subgroups).^1^ A previous study has shown in SEER-Medicare that men older than 65 years with limited LE owing to comorbidity burden exhibit significant early improvements in cancer-specific mortality with aggressive (vs conservative) treatment, with 5-year absolute risk reductions in cancer mortality of 7%, 5.5%, and 6.9% for men with Charlson Comorbidity Index scores of 0, 1, and 2, respectively (*P* < .05 for all).^27^ Ideally, if men with limited LE would have had a more rapid decline in the use of RP for low-risk and favorable intermediate-risk PCa and a similar increase in the proportion treated for high-risk disease compared with men with greater longevity, the trends would better match risks with potential benefits associated with surgery.

There are numerous LE prediction tools that can be used to accurately estimate LE in the clinical setting, which may help reduce the mismanagement of men with limited LE.^17,28,29,30^ Although most practitioners rely on actuarial life tables based solely on age to estimate LE, doing this ignores health status, which other studies have shown has a strong independent association with long-term, other-cause mortality.^14,30^ The National Comprehensive Cancer Network guidelines recommend adding or subtracting 50% of life-years based on whether a patient is in the highest or lowest quartile of health, but there is no reliable method of determining who is in the higher or lower quartile, and there is significant heterogeneity in survival among those in the lower quartile.^31^ Therefore, the PCCI—an LE prediction tool based on a weighted scale of age and comorbidities—was developed to predict long-term, other-cause mortality in men with PCa.^17^ This concept has been extrapolated to use of a count of comorbidities and has been optimized for clinical use in the form of life tables.^28^ Other groups have created population-based LE prediction tools based on age and comorbidity,^30^ and yet others have created treatment-specific tools.^29^

### Limitations

Several limitations of the current study are worth noting. First, the study sample consisted entirely of patients with PCa treated at 8 VA medical centers. Therefore, the results of the present study may not be readily generalizable to health care systems outside of the Veterans Health Administration, which have different payment models and patient sociodemographic characteristics that may both impact treatment choice. Second, the freedom to seek medical care outside of the Veterans Health Administration system may have led to the misclassification of men into smaller PCCI score subgroups owing to incomplete VA medical records on comorbidity status; this scenario could potentially diminish the difference in trends between PCCI subgroups. Third, we did not have a sufficient sample size to investigate differences in RP trends among the 8 VA medical centers. Immense variation in the primary treatment of localized PCa at both the clinical level and clinician level^26,32^ emphasizes the need for further studies on variation of cancer treatments in an effort to address discrepancies, improve treatment decision supports, and establish best-practice standards that align with national clinical practice guidelines.

## Conclusions

In this study, we found that the tumor risk case mix among men receiving RP in the VA health care system has drastically changed in the active surveillance era, with increasing use of RP for high-risk and unfavorable intermediate-risk tumors and dramatically decreasing use for low-risk tumors. Despite these changes, there is little difference in treatment trends by LE across tumor risk subgroups, and there is an overall increase in the use of RP among men with limited LE. Ideally, we would see sharper decreases in rates of RP use for lower-risk cancers and similar increases for high-risk cancers among those with limited LE, which were not observed in our data. Although the active surveillance era has shifted treatment paradigms based on tumor risk, there appears to be a persistent problem with appropriate management of men with limited LE. Adherence to guidelines and use of validated tools to predict LE may assist in improving management of these men.

## Notes

Supplement. **eFigure.** Use of Radical Prostatectomy Stratified by Tumor Risk and PCCI Scores
