# Supplementary material for: Time Trends in Use of Radical Prostatectomy by Tumor Risk and Life Expectancy in a National Veterans Affairs Cohort
Source: JAMA Netw Open. 2021 Jun 3;4(6):e2112214. doi: 10.1001/jamanetworkopen.2021.12214 (PMC8176332; doi:10.1001/jamanetworkopen.2021.12214)
Supplement: Supplement. — eFigure. Use of Radical Prostatectomy Stratified by Tumor Risk and PCCI Scores [file jamanetwopen-e2112214-s001.pdf]

# Supplementary Online Content

Vaculik K, Luu M, Howard LE, et al. Time trends in use of radical prostatectomy by tumor risk and life expectancy in a national Veterans Affairs cohort. *JAMA Netw Open.* 2021;4(6):e2112214. doi:10.1001/jamanetworkopen.2021.12214

**eFigure.** Use of Radical Prostatectomy Stratified by Tumor Risk and PCCI Scores

This supplementary material has been provided by the authors to give readers additional information about their work.

© 2021 Vaculik K et al. *JAMA Network Open.*

**eFigure.** Use of Radical Prostatectomy Stratified by Tumor Risk and PCCI Scores

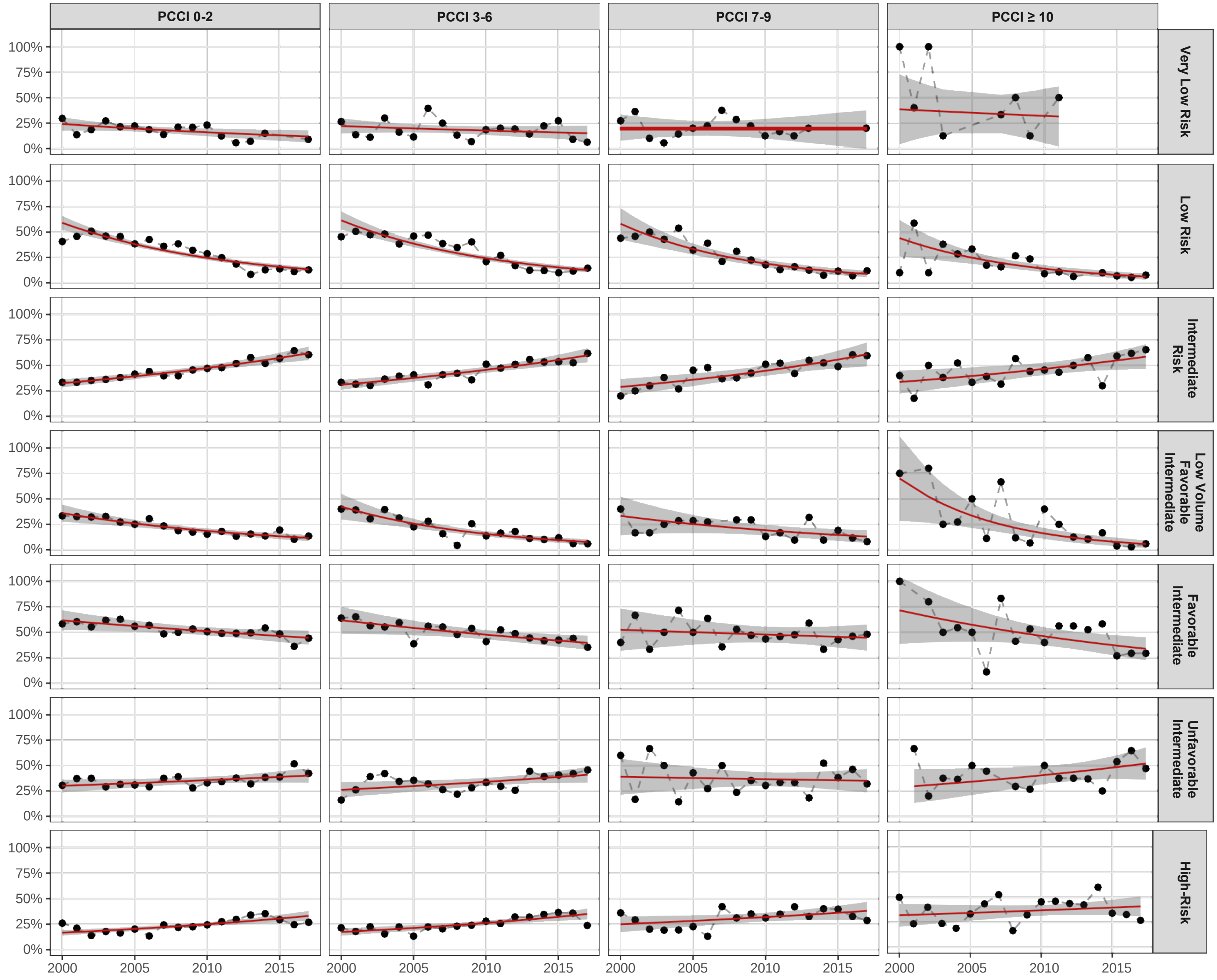


Points and dashed line indicate raw values and unadjusted trend for proportion of men treated with RP over time
Red line indicates the estimated proportion of men treated with RP over time using the log-linear Poisson regression model
The shaded area represents the 95% confidence interval of the estimates from the log-linear Poisson regression model

© 2021 Vaculik K et al. *JAMA Network Open.*
